# Relating traditional and academic ecological knowledge: mechanistic and holistic epistemologies across cultures

**DOI:** 10.1007/s10539-018-9655-x

**Published:** 2018-11-22

**Authors:** David Ludwig, Luana Poliseli

**Affiliations:** 1 Knowledge, Technology, and Innovation (KTI) Group, Wageningen University, Hollandseweg 1, 6706 KN Wageningen, The Netherlands; 2 Laboratório de Ensino, Filosofia e História da Biologia, Instituto de Biologia, Universidade Federal da Bahia, Rua Barão de Jeremoabo, 668, Salvador, Bahia 40170-115 Brazil

**Keywords:** Traditional ecological knowledge, Indigenous knowledge, Mechanisms, Mechanistic explanation, Holism, Philosophy of ecology

## Abstract

Current debates about the integration of traditional and academic ecological knowledge (TEK and AEK) struggle with a dilemma of division and assimilation. On the one hand, the emphasis on differences between traditional and academic perspectives has been criticized as creating an artificial divide that brands TEK as “non-scientific” and contributes to its marginalization. On the other hand, there has been increased concern about inadequate assimilation of Indigenous and other traditional perspectives into scientific practices that disregards the holistic nature and values of TEK. The aim of this article is to develop a practice-based account of the epistemic relations between TEK and AEK that avoids both horns of the dilemma. While relations between TEK and AEK are often described in terms of the “holistic” nature of the former and the “mechanistic” character of the latter, we argue that a simple holism–mechanism divide misrepresents the epistemic resources of both TEK and AEK. Based on the literature on mechanistic explanations in philosophy of science, we argue that holders of TEK are perfectly capable of identifying mechanisms that underlie ecological phenomena while AEK often relies on non-mechanistic strategies of dealing with ecological complexity. Instead of generic characterizations of knowledge systems as either mechanistic or holistic, we propose to approach epistemic relations between knowledge systems by analyzing their (partly mechanistic and partly holistic) heuristics in practice.

## Introduction

Indigenous, local, and traditional ecological knowledge (IEK, LEK, TEK) are increasingly recognized in the biological and environmental sciences but also raise fundamental methodological questions about the limits of knowledge integration. On the one hand, Indigenous scholars have emphasized the importance of TEK for understanding and managing local environments (Cajete 2000; Pierotti and Wildcat 2000; Wilson 2008) and have contributed to growing debates about “co-creation”, “co-management”, “participation”, and “transdisciplinarity” (Berkes 2018; Chapman et al. 2016; Hadorn et al. 2006; Parsons et al. 2016) in areas such as agriculture and conservation. By attacking what Agrawal (1995) has called the “divide between Indigenous and scientific knowledge”, these accounts typically combine epistemic and political concerns (Whyte 2018). Holders of TEK are epistemically recognized as experts (Byskov 2017) about local environments and this expertise is mobilized to challenge their political marginalization (Scott 1998; Shepherd 2006; White 2014) in negotiations of practice and policy.

On the other hand, attempts to integrate TEK into institutionalized science often create tensions with its definitional feature of being part of the heritage of specific communities. While there is a well-established academic consensus about the relevance of TEK in the biological and environmental sciences, the line between integration and assimilation is often far from clear. For example, El-Hani and de Ferreira Bandeira (2008: 7) warn that emphasis on the scientific character of TEK can lead to a loss of “the distinctiveness of other ways of knowing, and, also, their epistemic value in terms of their own validation criteria.” Related concerns are articulated in Nadasdy’s (2003) influential critique of integration projects that run the risk of extracting “scientifically useful” information from TEK while marginalizing (e.g. ethical and spiritual) aspects that do not fit the agendas (Castleden et al. 2017), methods (Marlor 2010), and ontologies (Ludwig 2018) of scientists.

The result seems to be a dilemma of division and assimilation in which either the epistemic potential or unique character of TEK becomes misrepresented through inadequate framing. For example, consider projects that aim to integrate TEK of Indigenous hunters with academic ecological knowledge (AEK)^1^ of conservation scientists. Indigenous hunters will often possess a lot of relevant expertise and may be best equipped to monitor vulnerable populations, identify causes of population decline, detect changes in ecological dynamics, identify requirements of sustainable hunting, and so on (Ens et al. 2015; Weiss et al. 2013). However, much of this expertise will also often be entangled with spiritual values and worldviews that diverge substantially from academic approaches to conservation management (Barbour and Schlesinger 2012). Treating holders of TEK as “collaborators” who can contribute evidence to AEK “just like scientists” therefore runs the risk of obscuring or completely missing core aspects of TEK.

The aim of this article is to develop an account that relates the epistemic resources of TEK and AEK while avoiding both horns of the dilemma of assimilation and division. Debates about the relations between TEK and AEK commonly start by characterizing the former as “holistic” and the latter as “reductionistic” or “mechanistic” (Aikenhead and Ogawa 2007; Dods 2004; Wood 1999). While such characterizations can convey important insights, this article argues that a simple holism–mechanism divide misrepresents the epistemic resources of both TEK and AEK. The following section emphasizes that holders of TEK are perfectly capable of identifying mechanisms that underlie ecological phenomena. Using the literature on mechanistic explanations in philosophy of science, we reinterpret Lansing’s (1991) classical case study of rice farming in Bali and argue that the neglect of TEK about ecological mechanisms turned out to be both epistemically inadequate and politically problematic. While holders of TEK can identify ecological mechanisms, the second half of the article emphasizes that TEK also often employs strategies that are holistic in two distinct meanings of the term. First, TEK commonly involves a methodological holism that avoids decompositional analysis and rather addresses dynamics of ecosystems as “wholes”. Second, Indigenous scholars often characterize TEK as holistic in a broader sense that emphasizes metaphysical, spiritual, and ethical dimensions of interrelatedness and reciprocity. The conclusion returns to the issue of differences between TEK or AEK by developing both a critical diagnosis and a constructive proposal. We argue that the prevalence of mechanistic resources in TEK and of holistic strategies in AEK undermines a simple holism–mechanism divide. Instead, we propose a more fine-grained analysis of heuristics in TEK and AEK that provides resources for engaging with both similarities and differences in a more substantial way that can inform negotiations of ecological knowledge between diverse stakeholders.

## Identifying and managing mechanisms with TEK

While it has become widely agreed that there is no simple demarcation criterion that distinguishes TEK and AEK (Agrawal 2014; Drouin-Gagné 2014; Gorelick 2014; Whyte 2013), acknowledgment of substantial differences between knowledge systems is often a requirement of adequate and respectful engagement with TEK. A common strategy of describing differences is to contrast the “holistic” nature of TEK with the “mechanistic” or “reductionistic” character of AEK or Western science more generally. Furthermore, this contrast often motivates a wider framing according to which “traditional knowledge [constitutes] a challenge to the positivist-reductionist paradigm in Western science” (Berkes 2018: 20) as it generates understanding of local environments and sustainable practices in a holistic way.

This article focuses on mechanisms rather than reductionism, as a generic characterization of Western science as “reductionistic” is clearly insufficient for a nuanced analysis of epistemic strategies in TEK and AEK. While reductions can be important epistemic strategies in scientific practice (Dizadji-Bahmani et al. 2010), it has been commonly noted in philosophical debates (Brigandt 2010; Kellert et al. 2006; Ruphy 2016) that science is often not reductionistic in practice. Furthermore, limitations of reductionist perspectives have been especially prominent in the life sciences (Brigandt and Love 2017; Kaiser 2015) and debates about different levels of organization have even been characterized in terms of an “anti-reductionist consensus” in philosophy of biology (see Waters 1990; Griffiths and Stotz 2013).

While a general characterization of AEK as reductionistic would be based on an simplistic picture of “Western science”, a focus on mechanisms constitutes a more promising starting point. Mechanistic approaches have been extraordinarily influential in recent philosophy of science, in part because they are framed as an alternative to traditional reductionist programs (Andersen 2014; Craver 2007; Fehr 2004). While many “New Mechanists” (Levy 2013; Franklin-Hall 2016) reject traditional reductionism and stress the importance of components across multiple levels of organization, they still focus on explanatory strategies that can be plausibly contrasted with holistic perspectives in TEK. Most importantly, mechanistic approaches involve decompositional analyses of “parts whose activities and interactions are responsible for the phenomena” (Illari and Glennan 2017: 1). Even if mechanistic philosophers do not assume that a target phenomenon can be completely reduced to a lower level of composing parts, the mechanistic focus on interacting sub-components suggests an attractive contrast with holistic perspectives on ecosystems “as wholes” that are commonly emphasized in TEK.

For example, an academically trained ecologist may analyze a target phenomenon such as the spread of an agricultural pest in terms of a mechanism of interacting components (e.g. intensification and monocropping that create a favorable habitat for a pest, loss of biodiversity that reduces prevalence of parasites and predators of the pest, development of pesticide resistances). In contrast, holders of TEK may employ pest management strategies (e.g. ethical and spiritual requirements of engagement with a forest, agricultural norms regarding polyculture or cropping schedules) in their agroecological systems that are effective even if they do not aim for an explanation of how different components interact in creating pest vulnerability. This way, both TEK and AEK can guide effective strategies of pest management even if they employ very different epistemic strategies.

While this contrast between holistic and mechanistic approaches has some prima facie plausibility, it provides an overly simple picture of the epistemic resources of both TEK and AEK. This section develops the argument that a limitation of mechanistic analysis to AEK obscures the capacities of TEK to identify mechanisms of complex ecological phenomena. Holders of TEK are not only capable of identifying mechanisms of complex ecological dynamics but also of intervening in them through equally complex management strategies. Taking the epistemic resources of holders of TEK seriously therefore also requires to recognize that the identification of mechanisms is not exclusive to AEK or Western science more generally.

Developing a more adequate account of the scope of mechanistic perspectives in TEK and AEK requires some specifications of what is meant with “mechanism”. Mechanism talk has become ubiquitous in recent philosophy of science and is used to develop heterogenous frameworks and with very different agendas (Levy 2013; Nicholson 2012). In its most ambitious interpretation, mechanistic perspectives are associated with what Dijksterhuis (1961) famously described as the “The Mechanization of the World Picture” in early modern science in Europe. Given such an appeal to what Nicholson (2012) calls “mechanicism”, it is not only obvious that TEK is non-mechanistic but also questionable whether current trends in AEK can be adequately described as mechanistic (Berkes 2018). However, substantial parts of mechanistic philosophy of science focus on specific epistemic strategies rather than these more general debates about “mechanistic worldviews”. For example, Machamer et al.’s (2000) landmark article develops a mechanistic framework with examples from molecular biology and neurobiology. Their guiding questions are what it means for molecular biologists to refer to the “mechanism of DNA replication” or for neurobiologists to refer to the “mechanism of chemical neurotransmission”. In both cases, Machamer et al. argue that scientists’ appeals to mechanisms reflect a distinct epistemic strategy that involves “entities and activities, organized such that they are productive of regular changes” (2000: 1). Applied to the relation between AEK and TEK, the question is therefore not whether holders of TEK subscribe to a “mechanistic worldview” (they don’t) but rather whether they also employ this distinctive epistemic strategy that has become commonly emphasized in scientific practice.

Illari and Williamson’s (2012) proposal of a general account of mechanisms helps to further specify these issues. While there has been little agreement on a general definition of “mechanism”, Illari and Williamson aim to provide a minimal account of core components by stating that a “mechanism for a phenomenon consists of entities and activities organized in such a way that they are responsible for the phenomenon” (2012: 120). This definition includes three components that can be illustrated with Machamer et al.’s examples of DNA replication and chemical neurotransmission: First, mechanisms are composed of entities and activities. For example, the mechanism of DNA replication is composed of entities such as the double helix and base pairs as well as activities such as the unwinding of former and bonding of the latter. The mechanism of chemical neurotransmission is composed of entities such as neurotransmitter molecules and post-synaptic neurons as well as activities such as the release of the former and the depolarization of the latter. Second, mechanisms require the organization of these entities and activities into a mechanism. Both DNA replication and chemical neurotransmission are clearly not produced by an unordered set of entities and activities but rather by their intricate organization. Third, entities and activities have to be organized so that they are responsible for the target phenomenon. For example, organized entities such as the double helix and base pairs and activities such as their unwinding and bonding constitute a mechanism that is responsible for the target phenomenon of DNA replication. Target phenomena and their mechanisms stand in clear relations of dependency in the sense that the former are responsible for the latter.

Illari and Williamson’s account of mechanisms is intended to be broadly applicable in scientific practice for case studies from social psychology to astrophysics. It expresses the assumption that researchers in very different disciplines share a broad epistemic strategy that analyzes the organization of entities and activities that are responsible for target phenomena. Given this broad characterization for application in different disciplines, it becomes possible to see how not only AEK but also TEK can involve mechanistic epistemic strategies. Holders of TEK are often not only aware of many of the entities and activities that are involved in ecological dynamics but also of how the specifics of their organization are responsible for a target phenomenon. Furthermore, it is often precisely this intimate knowledge of entities, activities, and their organization that puts holders of TEK in the position to manage local environments by intervening in local mechanisms. The following section develops these claims in the context of Lansing’s (1991) classical case study of TEK about rice farming in Bali and of the marginalization of this knowledge in the wake of the Green Revolution.

### Organizing mechanisms through water temples

Lansing’s (1991) work on pest control and water management in Balinese rice farming has become widely recognized as a classical case study both of the complexity of TEK and of its marginalization for the sake of allegedly rational agricultural modernization. As Lansing emphasizes in the introduction of his *Priests and Programmers,* his research in Bali was originally designed as a much more traditional anthropological study of the historical development of Balinese temples but the “fieldwork happened to coincide with a phenomenon that seemed at first to have nothing whatever to do with temples: the onset of the ‘Green Revolution’ in Balinese agriculture” (1991: 3). The Green Revolution swept Indonesia in the early 1970s enforcing dramatic changes in rice farming in Bali such as the replacement of traditional varieties of rice with new cultivars that promised quicker maturation and higher yields. Achieving these yields did not only require new seeds but also a fundamental transformation of agricultural practices that mandated intensive use of fertilizers and pesticides as well as the abandonment of traditional cropping and irrigation patterns. While these patterns used to be determined by communities in the context of religious ceremonies of water temples, the new system mandated continuous rice cropping with a new cycle starting immediately after the harvest.

As Lansing documents in detail, this new system had disastrous consequences for local farmers and threatened their livelihoods by leading to water shortages and pest outbreaks: The rice variety IR-8 turned out to be susceptible to the brown planthopper and massive crop damage motivated a shift to the planthopper-resistant variety IR-36. However, IR-36 was affected by an explosion of the tungro virus that lead to the adoption of PB-50 which was affected by an outbreak and rapid spread of *Helminthosporium oryzae*. “Thus by the mid-1980s, Balinese farmers had become locked into a struggle to stay one step ahead of the next rice pest by planting the latest resistant variety of Green Revolution rice” (Lansing 1991: 115). Against the backdrop of this modernization failure, Lansing’s investigation of Balinese TEK focused on the role of water temples in coordinating a complex system of irrigation and cropping. His analysis of water management and pest control provides a clear case of the capacity of TEK to identify mechanisms of complex ecological dynamics and to intervene in these mechanisms through adaptive management strategies. It is therefore hardly surprising that the Balinese system of water management and pest control in rice farming can be understood along Illari and Williamson’s three criteria of (1) entities and activities, (2) organization, (3) responsibility for the target phenomenon.

*First,* traditional Balinese rice farming involves a range of entities and activities that can be described as constituting a mechanism for water management and pest control. Important entities include water temples and subaks, i.e. groups of roughly a hundred rice farmers who share water from a common source. Other relevant entities include rivers, canals, mountains, rice fields, pests, rice varieties, pesticides, villages, priests, Green Revolution engineers, and the regional government. Many relevant activities and processes can be identified in relation to these entities. For example, seasonally variable flow of water in the main rivers, the use of temples to coordinate the flow of water in partly synchronized irrigation and fallow patterns, scheduling of cropping activities, spraying of pesticides, and government mandates for certain agricultural activities.

*Second,* water management and pest control needs to be understood through the organization of these entities and activities into coordinated (or dysfunctional) systems. Indeed, water flows in Bali had been carefully engineered and organized through Balinese TEK. One of Lansing’s core findings was that pest control through Balinese TEK was largely achieved through synchronization of fallow periods that prevented the spread of pests such as rodents and insects as well as bacterial and viral diseases. By replacing the coordinating functions of water temples with a system of immediate replanting after the harvest, Green Revolution engineers created a desynchronized cropping schedule in which “migrating pests moved across the landscape consuming one harvest after another” (Lansing 1991: xxii). However, synchronization of irrigation and fallowing schedules has its limitations in Bali as synchronization across all subaks would create water shortages during peak irrigation demands. Water temples therefore turned out to have a very complex task of fine-tuning the water flow in a way that creates sufficient synchronicity to control pests but sufficient variation in irrigation schedules to avoid water shortage.

*Third*, Lansing provides strong evidence that the organization of these entities and activities is actually responsible for the target phenomena of (successful or dysfunctional) water management and pest control. In the case of the traditional Balinese system of rice farming, Lansing develops a detailed description of how sub-components such as irrigation schedules and fallow periods allowed effective water sharing during the dry season and pest control. In the case of novel Green Revolution practices, he shows how pest spread and water shortages were caused by undermining core components of the old mechanisms and its replacement with entirely desynchronized cropping schedules. In this sense, there is a clear case that Illari and Williamson’s criterion of “responsibility for the phenomenon” is satisfied.

In addition to this qualitative evidence, a key component of Lansing’s *Priests and Programmers* is a computer simulation that confirms the responsibility of traditional irrigation schedules for creating an adequate balance between pest spread and water stress. Along the Oos and Pentau rivers, farmers were divided into 172 subaks that formed 14 organizational units through water temples that produced 14 different irrigation schedules. Based on data on issues such as seasonal rainfall, water demand of subaks, water stress effects on plants, and pest spread, Lansing’s model predicts effects of different synchronization scenarios on water stress and pest spread. There are clear risks of both over- and under-synchronization. If all 172 subaks follow the same irrigation schedule, water stress reaches a maximum. If all 172 subaks follow individualized irrigation schedule (as promoted in the Green Revolution), pest damage is maximized. The predictions of the model converge with the actual organization of the temple system as 14 internally synchronized schedules provide an optimal balance by minimizing water stress and keeping pests at a comparatively low level. In this sense, the computer model provides further evidence that the water temple system constitutes a carefully engineered mechanism that is responsible for the target phenomena of water management and pest control.

To sum up, Lansing’s analysis clearly demonstrates the capacity of Balinese TEK to identify as well as engineer complex ecological mechanisms that meet Illari and Williamson’s three criteria of (1) entities and activities, (2) organization, (3) responsibility for the target phenomenon. A limitation of Lansing’s analysis, however, is that it says relatively little about how Balinese holders of TEK identify these mechanisms when explaining ecological dynamics. Although Lansing’s own account of the Balinese temple system clearly fits common characterizations of mechanistic explanation (e.g. Glennan 2002), his discussion does not provide a comprehensive analysis of the explanatory strategies that are employed by Balinese holders of TEK. While a detailed account of such explanatory strategies in Balinese TEK is missing, substantial research on causal reasoning has been developed in other contexts of TEK. For example, Atran and Medin’s group has engaged with TEK of Itza’ Maya and shown how ecological expertise of Itza’ grounds causal reasoning in terms of specific ecological mechanisms rather than more generic explanatory strategies (Medin and Atran 2007; Bailenson et al. 2002).

### The epistemology and politics of identifying mechanisms in TEK

The hypothesis that the identification of mechanisms is an exclusive characteristic of AEK has some intuitive appeal that is largely based on the common contrast between the holistic nature of TEK and the mechanistic orientation of Western science. However, case studies such as rice farming in Bali show that this hypothesis is simply false. The Balinese system of water management and pest control does not only illustrate the capacity of Balinese TEK to identify mechanisms of complex ecological dynamics but also its ability to engineer these dynamics through complex and multi-generational interventions in the mechanisms that produce them.

One of the strengths of Lansing’s analysis is that it does not only illustrate the resources of TEK to identify mechanisms that underlie complex ecological dynamics but also the epistemic and political costs of their neglect. Engineers of the Green Revolution simply did not recognize the mechanisms that were organized through water temples and Lansing leaves little doubt that this failure was embedded in a more general disregard for Balinese TEK: “The answer to pests was pesticide, not the prayers of priests. Or as one frustrated American irrigation engineer said to me, ‘These people don’t need a high priest, they need a hydrologist!’” (Lansing 1991: 115).

Disregard for Balinese TEK came with entangled epistemological and political costs. On the epistemological side, the insufficient recognition of mechanisms that were embedded in TEK resulted in a predictive failure in the sense that Green Revolution engineers were unable to anticipate and to respond to water shortages and pest outbreaks. Politically, this failure was disastrous for Balinese farmers whose harvests were ruined by paternalistic policies that did not even understand what they were destroying. As Lansing (1991: 161) describes the situation: “the threat of legal penalties against anyone failing to grow the new rice led to continuous cropping of Green Revolution rice. Religious rituals continued in the temples, but field rituals no longer matched the actual stages of rice growth. As soon as one crop was harvested, another was planted, and cropping cycles began to drift apart. During the rainy season, no one was likely to run out of water. But during the dry season, the supply of irrigation water became unpredictable. Soon, district agricultural offices began to report ‘chaos in the water scheduling’ and ‘explosions of pest populations,’ as in this 1985 report by the Department of Public Works of the regency of Tabanan.”

While Lansing’s account of the Green Revolution in Bali can be situated in wider controversies about the social effects of agricultural intensification and modernization (Conway and Barbier 2013; Holt-Giménez and Altieri 2013; Horlings and Marsden 2011), our analysis aims for a more restricted political lesson. No matter whether goals of increasing crop yields through agricultural intensification were justified, the neglect of epistemic resources of Balinese TEK in the intensification process led to adverse effects on the livelihoods of local communities. An overly simple contrast between an exclusively holistic perspective of TEK and an exclusively mechanistic perspective of AEK is therefore not only epistemologically inadequate but can also come with very concrete political costs for holders of TEK. In other words, the idea that mechanism discovery is exclusively the business of Western science and nothing to do with the holistic worldview of TEK runs the risk of producing entangled epistemic misrepresentations and social injustices by disregarding the expertise of local communities and embracing harmful practices.

## Limitations of mechanistic approaches in TEK

In a collaborative project that integrates ecology and philosophy of science, Poliseli (2018) developed a detailed analysis of the potential of mechanistic approaches in AEK. Analyzing an autochthonous bee community in an agricultural pollination service in Brazil, Poliseli et al. emphasize the guiding role of heuristics in the construction of their mechanistic model. Understanding heuristics broadly as epistemic tools that guide engagement with target phenomena, the project developed a diverse toolbox of heuristics that was employed to generate an account of the pollination service in terms of underlying mechanisms. For example, Poliseli et al.’s heuristics include *phenomenon characterizations* that specify explanans and explanandum, *mechanism sketches* that visualize relations within the pollination service, *hierarchical structures* that locate components of the explanans at different levels of organization, and *enabling condition*s that identify core variables of the mechanisms in the pollination service.

Given the arguments from the last section, it will not surprise that some of Poliseli et al.’s heuristics are not restricted to AEK but can also be applied in contexts of TEK. For example, the complex Balinese system of rice farming requires identification of core *enabling conditions* such as irrigation and cropping schedules and tools of visualization have been widely documented in TEK and Indigenous knowledge even if they are not formally structured as *mechanism sketches* (Joyce 2012; Begossi and Caires 2015). That being said, Poliseli et al. developed their heuristics to guide the development of a specific mechanistic model for a specific ecological phenomenon and does not suggest that their heuristics will be applicable in all contexts of AEK or even TEK.

While some TEK heuristics can contribute to a better understanding of mechanisms, this section argues that many TEK heuristics are valuable precisely because they circumvent questions about the organization of entities and activities that is responsible for a target phenomenon. Mackinson’s (2000) influential study of local knowledge of fishery in British Columbia provides a helpful starting point for developing the argument that avoiding mechanistic perspectives is a productive feature of many epistemic strategies in TEK. Based on interviews with First Nations, non-indigenous fishers, fishery managers, and fishery scientists, Mackinson collected a large set of IF … THEN clauses about the behavior of herring shoals and integrated them in the expert system CLUPEX. Mackinson refers to these IF … THEN clauses as heuristics and emphasizes the expertise of local informants in identifying them. Examples of locally communicated heuristics include: “If there is a bright moon the fish spread out fast and it is difficult to catch them” and “If weather is bad close to spawning then schools get disrupted”. Heuristics in Mackinson’s case study share the feature of relating specific attributes such as bird predator type, current strength, moonlight, water depth, or weather with equally specific descriptors of herring shoals such as size, packing density, depth, shape, cohesion, and movement of the shoal. If such relations are consistently and robustly communicated across several informants, they are translated into more formalized IF … THEN clauses such as “IF time of day is dusk OR night AND state of the moon is full and bright, THEN shoal depth mid-range AND packing density very low AND ease of capture very low” (Mackinson 2000: 540).

Mackinson’s and Poliseli et al.’s heuristics differ as the former aim at dynamics of ecosystems at the macro-level without providing an account of underlying mechanisms. Poliseli et al.’s heuristics address pollination by developing a detailed account of the organization of entities and activities that are responsible for the target phenomenon. Their case study therefore illustrates how AEK can be mechanistic in the sense of Illaris and Williamson’s (2012) criteria from the last section. In contrast, Mackison’s heuristics do not satisfy Illari and Williamson’s core criterion of responsibility as they identify descriptors that can be used as indicators for the target phenomenon of herring behavior but do not include assumptions about how (and whether) the former is responsible for the latter. Of course, some informants may combine descriptors such as moon phase or weather conditions with accounts of their causal contribution to the target phenomenon. However, the successful application of the heuristics does not require such accounts and can also be embedded in belief systems that develop very different explanations of regularities. For example, a heuristic that relates brightness of the moon and packing density of herring shoals could be effective while being embedded in very different causal stories in TEK that strongly differ from AEK by being framed in terms of intentional actions of the moon.

Mackinson’s case illustrates the importance of TEK that is not mechanistic in character because it aims for patterns and regularities at the macro-level without attempting to explain how a target phenomenon is produced through the organization of sub-components. The importance of this knowledge about dynamics of ecosystems at the macro-level is acknowledged by Mackinson and constitutes his main motivation for integrating local knowledge in AEK about herring shoals. Given “our incomplete understanding of biological and ecological mechanisms underpinning behavioral responses of fish” (Mackinson 2000: 533), it is often not feasible to build predictive models exclusively on the basis of mechanistic analyses. At the same time, predictive modeling is needed for purposes of conservation and fishery management. The expert system CLUPEX aims to solve this problem of insufficiently understood ecological complexity by circumventing questions about mechanisms and by building a predictive model on the basis of regularities and patterns at the macro-level.

Both the problem and the solution are well-known to holders of TEK whose livelihoods depend on interactions with complex ecosystems and who often have to make rapid decisions on how to interact with their environments during fishing and other daily activities. Intricate models of the organization of productive sub-components in the sense of Illari and Williamson can be feasible in some situations such as Balinese rice farming where TEK institutions invest considerable resources to monitor and reorganize relevant entities and activities such as dams and cropping schedules. However, this does not mean that mechanistic perspectives will always provide the most successful strategies for dealing with complex ecological systems in TEK. Heuristics that circumvent issues of underlying mechanisms will often be preferable in applied contexts such as conservation management and are widely employed in TEK.

### Holism in TEK heuristics

While Mackinson’s case study indicates limitations of mechanistic interpretations of TEK, it is less clear that it provides a substantive illustration of its holistic character. Indeed, the notion of holism is ambiguous and has been historically used to articulate a large variety of positions from strong anti-materialist theories to methodological programs that emphasize limitations of decompositional analysis without being committed to wholes as metaphysically distinct from their composing parts (Allen 2017). Holism in the more restricted methodological sense aims “to account for living processes as functioning wholes” (Allen 2017: 61) and rejects requirements of mechanistic analysis or even reduction in scientific practice (see also Ludwig 2012; Harrington 1999; Zahle and Collin 2014). Such a more restricted methodological interpretation of holism can also be applied to Mackinson’s case study as his examples illustrate that holders of TEK often address ecosystems “as functioning wholes” that exhibit relevant patterns and regularities while avoiding decompositional approaches of mechanistic or even reductionistic analysis.

Even if such a restricted methodological interpretation can be applied to Mackinson’s case study, “holism” in the context of TEK is commonly associated with wider metaphysical, spiritual, and ethical aspects of Indigenous worldviews. Characterizations of TEK as holistic do not only point to a methodology of investigating ecosystems as “functioning wholes” but rather to more general relational beliefs and practices of holders of TEK (Ingold 2006; Kovach 2010; Mika 2015; Shroff 2011). For example, Cajete’s influential *Native Science* characterizes Indigenous holism in terms of a general commitment to interrelatedness: “Humans are but one manifestation of an implicate universal order. All parts of this order interpenetrate one another. They are holistically codependent—‘we are all related.’’ (2000: 208). Appeals to interrelatedness in Indigenous holisms have entangled metaphysical, spiritual, and ethical meanings. “We are all related” is partly a causal claim about mutual influences but it is more than just propositional knowledge about connections between people and the land. For example, Ingold has influentially stressed relational perspectives in TEK as practices and skills of interacting with local environments: “For local people […] traditional knowledge is inseparable from actual practices of inhabiting the land. For it is in the relationships that are forged with the land, along with its animal and plant life, that their knowledge is generated” (Ingold and Kurttila 2000: 186).

Finally, relational perspectives do not only affect spiritual relations with the land from ancestral grounds to totemism (Viveiros de Castro 2012; Descola 2015) but are also materialized in moral practices of mutual responsibility and respect. As Shroff (2011: 53) puts it: “The holistic concept of social, psychological interconnectivity is a central aspect of Indigeneity. […] Concepts of interconnectivity within Indigenous ways of knowing translate into various values: relationships with others and self and ultimately to helping others and being of service.” Mackinson’s case study does not illustrate this wider interpretation of holism in the sense practices that emphasize metaphysical, spiritual, and ethical interrelatedness. On the contrary, his heuristics are clearly not holistic in this wider sense as they isolate propositional knowledge about very specific macro-regularities that allow modelling of equally specific ecological phenomena independently of Indigenous worldviews of interrelatedness.

While specific heuristics in the sense of Mackinson’s case study are important for holders of TEK in contexts such as sustainable fishing or forest management, it would be a misunderstanding to assume that the holism of TEK is exhausted by the identification of patterns and regularities of ecosystems “as functioning wholes”. On the contrary, characterizations of TEK as holistic typically reflect the entanglement of ecological knowledge with wider worldviews and practices of interrelatedness. To address the role of this wider holism in local heuristics, it is helpful to look beyond Mackinson’s case study of specific IF … THEN clauses. For example, Berkes’ discussion of Cree hunting practices builds on a much more in-depth attempt to situate epistemic practices in the Cree worldview “from the inside” (2018: 98). Berkes identifies three elements of Cree TEK that contrast with perspectives that are dominant in AEK. First, animals and not hunters control the hunt. Partly reflecting on the cyclical nature of animal abundance in Arctic and Subarctic regions (e.g. caribou, beaver, marten, porcupine), Cree emphasize the agency of animals in making (or not making) themselves available. Second, hunters and fishers are obligated to show respect to animals. Given the agency of animals in the hunting process, respect (e.g. in tracking, killing, butchering, and consuming) is a central requirement for engagement. Third, continuous use of resources is necessary for maintenance of hunting grounds. Excessive hunting and neglect of hunting grounds are equally harmful extremes that need to be avoided through continued and respectful forms of use.

Berkes’ analysis of Chisasibi Cree hunting of caribou emphasizes the importance of these general elements of Cree worldview in shaping rules of engagement. For example, issues of respect took the centre stage for Chisasibi Cree with the sudden reappearance and subsequent disappearance of large numbers of caribou of the George River herd. The population size of the George River herd has fluctuated between 5000 and 776,000 members through the twentieth century (Gunn et al. 2011) and reached a historic low by the end of the 1970s. Berkes focuses on a period in the 1980s that started with the return of a large number of caribou to the land of Chisasibi Cree in 1982–1983 but was followed by a sharp drop in 1984–1985 after an unregulated hunt that was perceived as violating the traditional agreements of respect between Chisasibi Cree and caribou. Chisasibi elders reacted to this development by reminding the community of its core rules and responsibilities. Many of these rules can be formulated as IF … THEN clauses. The caribou hunt is going to be successful again in the coming years only if, Chisasibi hunters (a) pay respect to the Caribou, (b) do not kill more Caribou than needed for use, (c) do not let carcasses rot, and so on.

Rules in Berkes’ case study may be called heuristics in the broad sense of epistemic tools that guide engagement with target phenomena. At the same time, these rules clearly differ from both Poliseli (2018) and Mackinson’s (2000) heuristics. Poliseli et al.’s heuristics are epistemic tools that generate ecological understanding by facilitating the identification of underlying mechanisms. Berkes’ rules have a very different structure as they guide sustainable practices without aiming for a mechanistic analysis. For example, a mechanistic model may attempt to specify the causal effects of different hunting practices on the population cycles and size of caribou herds. This would require differentiating the effects of hunting from other factors such as availability of winter food sources such as lichen, prevalence of other predators, harshness of winter, habitat destruction and pollution, and so on. While there has been some understanding of the contributions of these factors, Berkes points out that scientists have not been able to construct a predictive model and “caribou population increases and decreases are a scientific problem yet unresolved” (2018: 134). Despite the lack of mechanistic explanation of caribou population cycles, Chisasibi need to engage with these cycles (and many other complex ecological phenomena) in a sustainable way. General rules of engagement and respect provide a strategy for shaping sustainable interactions without requiring detailed knowledge of how the organization of different sub-components produces population cycles.

While Berkes’ (2018) and Mackinson’s (2000) heuristics can both be described as non-mechanistic, there are also important differences between them. Mackinson’s heuristics provide very specific IF … THEN clauses that isolate relations between equally specific ecological phenomena such as a weather condition and the packing density of herring shoals. In contrast, many rules in Chisasibi hunting are much more general in character and remain applicable where neither underlying mechanisms nor specific predictive IF … THEN clauses are available. Characterizing these rules as holistic is not only a matter of a particular epistemic strategy that focuses on dynamics of ecosystems at the macro-level rather than the organization of its sub-components. Instead, Chisasibi rules of hunting caribous are holistic in the sense that they are entangled with general elements of Chisasibi worldview and way of life. On the one hand, more general aspects of Chisasibi worldview such as the agency of animals shape the character of the rules that employed during hunting of caribou. On the other hand, rules of engagement with caribou and other animals have shaped Chisasibi worldview over the course of many generations. In other words, norms of hunting and general elements of Chisasibi worldview shape each other in a way that undermines attempts to isolate epistemic aspects from wider ethical, spiritual, and metaphysical components of Cree perspectives on environments. While philosophers of science often think of holism largely in terms of non-reductionistic or non-mechanististic methodologies, it is precisely this wider metaphysical, spiritual, and ethical entanglement that many Indigenous researchers have in mind when characterizing their perspectives as holistic (Cajete 2000; Wilson 2008). For the latter, the core is not the employment of a specific epistemic strategy but rather that the very divide between epistemic strategies and ways of life is artificial in TEK.

To sum up, this section has argued that holders of TEK employ a diverse toolbox of mechanistic and non-mechanistic heuristics. Thinking of heuristics broadly as epistemic tools that guide engagement with target phenomena, different ecological case studies lead to different types of heuristics. The last section emphasized that holders of TEK can identify and intervene on mechanisms but not all heuristics in TEK contribute to mechanistic accounts of the organization of entities and activities that are responsible for target phenomena. First, Mackinson’s (2000) discussion of local fishing knowledge documents heuristics that relate ecological phenomena at the macro-level without requiring decompositional accounts of mechanisms. Second, Berkes’ (2018) discussion of Chisasibi Cree TEK documents heuristics that guide hunting independently of the identification of underlying mechanisms or specific macro-regularities. TEK therefore includes highly heterogeneous set of mechanistic and holistic heuristics that can guide engagement with local environments under diverse epistemic and pragmatic conditions.

## Conclusion: relating knowledge systems beyond the mechanism–holism divide

This article started with the common criticism of a “divide between Indigenous and scientific knowledge” (Agrawal 1995) as creating artificial boundaries and thereby contributing to the marginalization of the former. To some degree, our discussion can be framed within this wider critique as it suggests two shortcomings of a simple mechanism–holism divide between TEK and AEK. First, generic characterizations of TEK as holistic obscure the diversity of its epistemic resources. Holders of TEK are perfectly capable of identifying and intervening in mechanisms of complex ecological phenomena such as the agroecological system of Balinese rice farming. Second, holistic elements are not exclusive to TEK and a characterization of AEK as entirely mechanistic also runs the risk of obscuring the diversity of its epistemic resources. At least holism in the more restricted methodological sense often plays an important role for successful engagement with complexity and uncertainty in AEK. Ecological phenomena are commonly shaped by a large number of insufficiently understood variables and feedback loops that limit the prospects of predictive modelling through mechanistic analysis. This is explicitly acknowledged both in Mackinson’s (2000) and Berkes’ (2018) incorporation of TEK into AEK and converges with more general debates about the limitations of mechanistic perspectives in philosophy of biology (Braillard and Malaterre 2015; Dupré 2013; Halina 2017; Woodward 2013). In other words: Mechanistic approaches are relevant in TEK and AEK without exhausting the epistemic resources of either of them.

Recognizing the inadequacy of a simple mechanism–holism divide matters for entangled epistemological and political reasons. Epistemologically, a simple contrast between holistic TEK and mechanistic AEK obscures the complexity of both knowledge systems in practice. In contrast with such a simple divide, this article has aimed to develop a more adequate account that acknowledges mechanistic and non-mechanistic features of epistemic practices beyond generic labeling of TEK and AEK as either holistic or mechanistic. While our discussion has mostly focused on these epistemological issues, it is important to acknowledge their entanglement with applied and political questions about the status of TEK. First, bringing the resources of mechanistic philosophy of science into debates about TEK is not only theoretically intriguing but helps to clarify epistemic resources that have often been neglected and led to the marginalization of holders of TEK in practice. Lansing’s Balinese case study provides a powerful illustration of this practical significance as the neglect of Balinese TEK in the wake of the Green Revolution led to pest outbreaks and water shortages with catastrophic consequences for local farmers. Second, it is equally important to avoid a reduction of TEK to its mechanistic resources as substantial parts of TEK cannot be isolated from the wider worldview and way of life local communities. Taking TEK seriously therefore also requires to look beyond its potential contribution to mechanistic analysis and to recognize its entanglement with holistic reasoning in TEK.

While this article has criticized a simple mechanism–holism divide, our introduction contrasted the problem of division with a problem of assimilation that downplays the unique (cultural, ethical, metaphysical, methodological, social, spiritual…) aspects of TEK for the sake of integration with AEK (El-Hani and de Ferreira Bandeira 2008; Ludwig 2016; Nadasdy 2003). And indeed, the analysis of this article is not limited to a rejection of the mechanism–holism divide but can also be used for a more fine-grained description of differences between epistemic resources in TEK and AEK. Rather than using generic characterizations of knowledge systems as “holistic” or “mechanistic”, we have argued for a heterogenous toolbox of heuristics with diverse mechanistic and holistic features. Although this general analysis applies to both TEK and AEK, a more detailed analysis of the application of heuristics in practice will also uncover substantial differences.

Even if both AEK and TEK can involve the identification of mechanisms, heuristics in AEK are commonly shaped by methodological requirements and standards of scientific research that strongly differ from those employed in TEK. For example, Poliseli (2018) heuristic of Evidence Frequency aims to identify causal relations between components on the basis of probabilistic and mechanistic evidence from various sources. While it may be possible to locate this general epistemic strategy in some contexts of TEK, Poliseli (2018) also specify this heuristic in terms of strategies such as analytic and synthetic analysis, forward and backward chaining, graphs and indexes that are largely specific to academically institutionalized research. Speaking more generally, heuristics in AEK are shaped by methods and standards (e.g. of experimentation, modelling, and quantification) that clearly differ from methods and standards that shape practices in TEK (see also Marlor 2010). One does not need a general mechanism–holism divide to acknowledge these differences in a more fine-grained account of the relations between epistemic strategies in AEK and TEK.

Furthermore, not only heuristics in AEK but also in TEK often involve unique aspects that need to be acknowledged in a more fine-grained comparison of epistemic strategies. For example, AEK commonly employs epistemic strategies that are holistic in the sense of addressing dynamics of ecosystems at the macro-level while avoiding mechanistic decomposition in terms of productively organized entities and activities. However, we argued in the context of Berkes’ case study of Caribou that many heuristics in TEK need to be understood as holistic in much wider sense of being entangled with the ethics, worldview, and way of life of Indigenous and other local communities. Of course, AEK is also entangled with values and worldviews of Western researchers and wider issues of interrelatedness are not exclusive to TEK. At the same time, Berkes discussion makes it very clear that many Cree heuristics are very different from heuristics that are employed by AEK as they are shaped by unique metaphysical beliefs (e.g. animals deciding to be caught) and values (e.g. how to respect a caribou) of Chisasibi Cree worldview. Even if AEK is not exclusively mechanistic, holistic heuristics in TEK will often be clearly distinguished from AEK heuristics in how they integrate metaphysical, spiritual, and ethical commitments of traditional communities.

The rejection of a simple mechanism–holism divide does therefore not collapse differences between AEK and TEK but provides opportunities to locate differences in more specific practices. Holders of AEK and TEK both employ diverse epistemic toolboxes that include mechanistic and holistic strategies but that does not imply that they are using identical strategies. Locating both similarities and differences in specific practices beyond a simple mechanism–holism divide is not only an interesting theoretical task for philosophy of science but can contribute to epistemologically and politically more adequate practices of co-creation. Understanding similarities (both through mechanistic resources of TEK and holistic aspects of AEK) is important for finding common ground in co-creation and avoiding an artificial divide between knowledge systems. Understanding differences (both in quantitative and qualitative characteristics of heuristics) is important for taking the unique characteristics of TEK seriously and avoiding its inadequate assimilation into academic research.

## Footnotes

1 There is no terminological agreement about Indigenous, local, and traditional ecological knowledge (IEK, LEK, TEK) (e.g. see Ingold and Kurttila 2000 on problems with the label “traditional”) and there is even less clarity about contrasting labels such as academic, scientific, and Western ecological knowledge (AEK, SEK, WEK). While AEK is not the most common label, it has advantages over SEK or WEK for current purposes. In contrast with SEK, AEK avoids boundary disputes and does not definitionally exclude TEK from qualifying as scientific. Furthermore, we prefer AEK over WEK because the issues of this article reflect not so much Western origin but rather academic institutionalization of knowledge systems. Knowledge “in the West” can be deeply entangled with traditions of particular local communities in Europe (e.g. Svanberg et al. 2011) and North America (e.g. Nolan 2007). Furthermore, knowledge “outside of the West” can become institutionalized in academic contexts with little connection to the traditions of a specific community.
